# Artificial Intelligence–Assisted Screening for Patients With Diabetic Retinopathy and Age-Related Macular Degeneration in Family Medicine and Geriatric and Gerontology Care: Protocol for a Pragmatic Randomized Clinical Trial

**DOI:** 10.2196/91699

**Published:** 2026-07-06

**Authors:** Bo-I Kuo, Ting-Ann Wang, Teresa Cheng-Chieh Chu, Ding-Cheng Chan, Shao-Yi Cheng, Chia-Ti Tsai, Chu-Lin Tsai, Yi-Chia Lee, Chiuan-Jung Chen, Wei-Li Chen, John Tayu Lee, Chia-Ying Tsai, Patrick Yan-Tyng Liu, Chi-Yang Chang, Chia-Ter Chao, Jia-Horng Kao, Yi-Ting Hsieh, the Collaborators of AI Ophthalmology Research Group, Hsiao-Sang Chu

**Affiliations:** 1 Graduate Institute of Clinical Medicine, College of Medicine, National Taiwan University, Taipei, Taiwan; 2 Department of Ophthalmology, National Taiwan University Hospital, No. 7, Chung-Shan South Road, Taipei, 10002, Taiwan, 886 2-2312-3456 ext 265018; 3 Integrative Medical Data Center, Department of Medical Research, National Taiwan University Hospital, Taipei, Taiwan; 4 Department of Geriatrics and Gerontology, National Taiwan University Hospital, Taipei, Taiwan; 5 Superintendent Office, National Taiwan University Hospital Bei-Hu Branch, Taipei, Taiwan; 6 Department of Internal Medicine, National Taiwan University Hospital, Taipei, Taiwan; 7 Department of Family Medicine, College of Medicine, National Taiwan University, Taipei, Taiwan; 8 Division of Cardiology, Department of Internal Medicine and Cardiovascular Center, National Taiwan University Hospital, Taipei, Taiwan; 9 Department of Emergency Medicine, National Taiwan University Hospital, Taipei, Taiwan; 10 Department of Internal Medicine, College of Medicine, National Taiwan University, Taipei, Taiwan; 11 Information Technology Office, National Taiwan University Hospital, Taipei, Taiwan; 12 Institute of Health Policy and Management, College of Public Health, National Taiwan University, Taipei, Taiwan; 13 Department of Ophthalmology, Fu Jen Catholic University Hospital, Fu Jen Catholic University, New Taipei City, Taiwan; 14 School of Medicine, College of Medicine, Fu Jen Catholic University, New Taipei City, Taiwan; 15 Division of Cardiology, Department of Internal Medicine, Min Sheng General Hospital, Taoyuan, Taiwan; 16 Division of Gastroenterology and Hepatology, Department of Internal Medicine, Fu Jen Catholic University Hospital, New Taipei City, Taiwan; 17 Division of Nephrology, Department of Internal Medicine, National Taiwan University Hospital, Taipei, Taiwan; 18 Graduate Institute of Toxicology and Graduate Institute of Medical Education and Bioethics, National Taiwan University, Taipei, Taiwan; 19 Division of Nephrology, Department of Internal Medicine, Min Sheng General Hospital, Taoyuan, Taiwan; 20 Hepatitis Research Center, National Taiwan University Hospital, Taipei, Taiwan; 21 Department of Ophthalmology, National Taiwan University Hospital Hsin-Chu Branch, Hsinchu, Taiwan; 22 See Acknowledgments

**Keywords:** artificial intelligence, diabetic retinopathy, age-related macular degeneration, fundus photography, screening, randomized controlled trial, cost-effectiveness

## Abstract

**Background:**

Diabetic retinopathy (DR) and age-related macular degeneration (AMD) are 2 of the leading causes of vision loss worldwide. As population aging and diabetes prevalence increase, timely detection of these conditions has become essential. However, limited professionalism and insufficient training in ophthalmic screening among general medicine physicians may lead to delayed diagnosis and treatment. Artificial intelligence (AI)–assisted diagnostic tools may help to improve the screening of DR and AMD in routine clinical practice.

**Objective:**

This study aims to evaluate the clinical effectiveness and cost-effectiveness of AI-assisted fundus imaging for DR and AMD screening in adults with diabetes and older adults at risk of macular degeneration.

**Methods:**

This multicenter, 2-arm, parallel-group, open-label, individual-level randomized controlled trial and patient recruitment are performed at the settings of Family Medicine and Geriatric and Gerontology Care over 4 medical centers in Taiwan. Eligibility includes (1) diabetic individuals aged ≥20 years for DR screening, and (2) individuals aged ≥50 years for AMD screening. The study protocol has been approved by the ethics committees of all participating hospitals, and all participants will provide written informed consent.

**Results:**

The study was funded in September 2024, began on October 2, 2025, and is expected to be completed in December 2027. After the pilot implementation phase without randomization, participants will be randomized 1:1 into two groups: (1) AI-assisted screening, and (2) usual physician-only screening. The primary outcomes will include the detection rates (defined as participants with confirmed DR or AMD among all screened participants) and the positive predictive values (defined as participants with confirmed DR or AMD among those who tested positive). Cost-effectiveness analyses will be performed using data derived from the trial results.

**Conclusions:**

This study will provide robust evidence on the effectiveness of AI-assisted ophthalmic screening in improving patient eye health outcomes through timely screening and accurate early detection. This strategy may be cost-effective.

## Introduction

Diabetic retinopathy (DR) is one of the most common retinal vascular diseases and a microvascular complication of diabetes, affecting approximately one-third of patients with diabetes [1]. It primarily impacts the retinal microvasculature and leads to retinal hemorrhages, microaneurysms, lipid exudates, and, in severe cases, neovascularization [2]. DR is a leading cause of vision impairment and blindness [3]. As the global aging population increases, the number of older individuals with diabetes continues to grow, contributing to a rising prevalence of DR [4,5]. Age-related macular degeneration (AMD), which affects the macula in individuals aged >50 years and is a leading cause of irreversible blindness, has an overall prevalence rate of 8.69% [6]. Early-stage AMD features drusen and abnormalities of the retinal pigment epithelium, while late-stage AMD is classified into 2 forms: neovascular (wet or exudative) and nonneovascular (dry, nonexudative, or atrophic) [2,3].

Effective screening and prompt intervention are crucial for preventing permanent vision loss. However, patients with diabetes and older people often have limited awareness of DR and AMD, poor compliance with fundus checks, and delayed seeking medical help. As chronic disease management mainly takes place in general medicine clinics, where physicians might find interpreting retinal images challenging, timely referrals to ophthalmologists are crucial. Introducing a reliable screening tool in general medicine settings can increase access to fundus examinations, enhance early diagnosis, and improve treatment outcomes.

In recent years, artificial intelligence (AI) technology has led to the development of deep learning software apps for medical image recognition and analysis, providing diagnostic support to general frontline physicians [4]. AI has demonstrated accurate performance in analyzing images from the fundus photography [5-7]. Using AI-based detection, classification, and algorithms [8], these tools offer high diagnostic accuracy for diseases such as AMD [9,10], DR [11], and glaucoma [12]. To address the high demand for specialist expertise and manpower, AI-assisted diagnostic software based on fundus photography in Taiwan, such as VeriSee AMD and VeriSee DR (Acer Inc), has been shown to enable physicians to rapidly identify retinal diseases [13-15]. However, whether AI-assisted screening can outperform traditional physician-led screening in real-world settings, particularly in terms of patient outcomes, remains unclear [16]. Furthermore, whether this technology can improve cost-effectiveness by reducing medical expenditures and improving patients’ quality of life through early detection and treatment is a critical issue that must be established from a health insurance reimbursement perspective. To date, limited evidence from pragmatic randomized trials [17] has examined the integration of AI-assisted retinal screening into routine general medicine workflows, particularly with simultaneous evaluation of clinical effectiveness, referral patterns, and economic outcomes [7]. In addition, existing evidence has largely focused on DR, with comparatively less attention given to AMD [18,19]. Addressing these gaps is essential for informing real-world implementation and reimbursement decisions.

The aim of this study is to evaluate the effectiveness and cost-effectiveness of integrating AI-assisted retinal screening tools for DR and AMD into routine clinical practice through a pragmatic randomized controlled trial (RCT) embedded in routine clinical care. The hypothesis is that incorporating AI-assisted retinal screening into routine care may facilitate earlier disease detection, improve patient outcomes, and represent a cost-effective strategy, thereby providing robust evidence to support the real-world adoption of AI technologies across diverse clinical settings.

## Methods

### Study Design

This study adopts a 2-phase design. The first phase is a pilot feasibility study conducted to evaluate system integration, workflow functionality, and operational readiness of AI-assisted retinal screening within routine clinical practice. The pilot phase began on October 2, 2025, and is expected to last for approximately 6 months to 1 year.

The decision to proceed to the second phase will be guided by referral rate–based criteria, based on pragmatic randomized trials [20,21], and progression to the second phase is not guaranteed.

**Figure 1.**
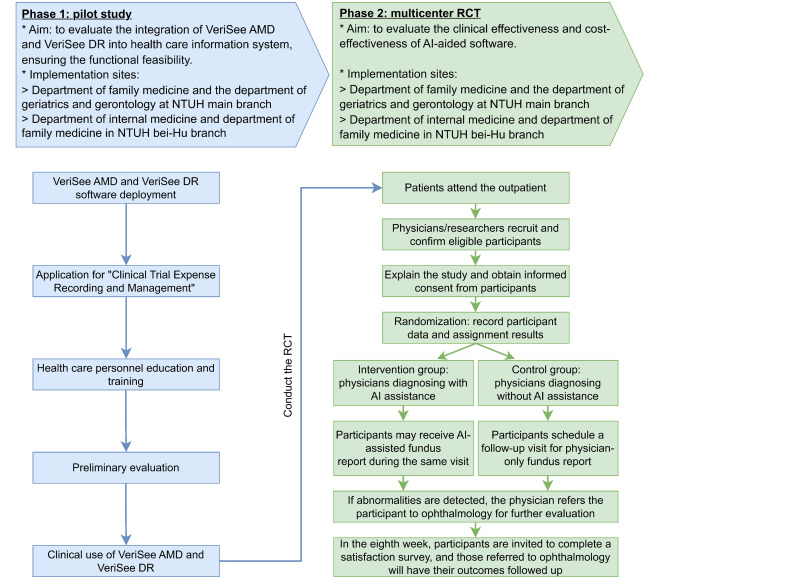
Illustration of study design. This figure illustrates the overall study design, including participant enrollment, informed consent, randomization to the artificial intelligence (AI)–assisted screening group or the conventional physician-based screening group, screening procedures, ophthalmology referral pathways, and follow-up for outcome assessment. AMD: age-related macular degeneration; DR: diabetic retinopathy; NTUH: National Taiwan University Hospital; RCT: randomized controlled trial.

The second phase is a multicenter, 2-arm, parallel-group, open-label pragmatic randomized clinical trial designed to evaluate the effectiveness of AI-assisted retinal screening compared with conventional physician-based screening. Owing to the nature of the intervention, blinding of the physicians performing the screening assessments is not possible. In addition, the ophthalmologists conducting the confirmatory diagnostic evaluations will not be blinded to group allocation, as the presence or absence of an AI-generated report may allow them to infer the assigned screening strategy. It reflects the real-world practice of using this AI instrument. Cost-effectiveness analyses will be conducted using trial data to simulate long-term economic benefits. The flowchart of this study is shown in Figure 1. The study followed the SPIRIT (Standard Protocol Items: Recommendations for Interventional Trials; Checklist 1) for RCT guideline [22].

### Study Settings

To ensure the performance of the AI screening test, a diagnostic accuracy study will be conducted concurrently with the RCT in the ophthalmology setting. Patients who visit the ophthalmology outpatient department will be invited to undergo the AI screening test without randomization, followed by confirmation of the diagnosis by the ophthalmology specialists. The reference standard will be diagnoses made by ophthalmologists. As both screen-positive and screen-negative individuals will undergo confirmatory assessment, sensitivity, specificity, and overall accuracy can be evaluated, which differs from the outcome assessment used in the randomized clinical trial (registration NCT06843499).

In the clinical trial, the study population will consist of adult patients attending family medicine and geriatric and gerontology outpatient clinics at the National Taiwan University Hospital (NTUH) Main Branch and Bei-Hu Branch, Fu Jen Catholic University Hospital, and Min-Sheng General Hospital in Taiwan.

### Inclusion and Exclusion Criteria

Inclusion criteria include adults aged ≥20 years with diabetes undergoing screening for DR and adults aged ≥50 years undergoing screening for AMD. Potential participants will be identified during routine clinic visits, and eligible individuals who express interest will receive detailed study information. Written informed consent will be obtained prior to enrollment. Participants will be excluded if they decline participation or are unable to provide informed consent because of cognitive or communication limitations.

### Screening Workflow

Eligible outpatient participants will be invited to participate in the study during routine clinic visits. After providing written informed consent, participants will undergo color fundus photography as part of the screening procedure.

For participants with fundus images, screening findings will be reviewed by the general medicine physicians. Participants without suspected retinal abnormalities will be advised to undergo routine follow-up according to standard clinical practice, whereas participants with suspected retinal abnormalities will be referred to ophthalmology for confirmatory evaluation. Ophthalmologic diagnoses will be established by ophthalmologists, and these will serve as the gold standard for determining true disease status and for calculating the detection rate and positive predictive value (PPV) of DR and AMD screening. Referral completion and diagnostic outcomes will be recorded, including cases in which referred participants did not attend the ophthalmology visit. Outcomes will be classified as confirmed or nonconfirmed retinal disease based on ophthalmologic assessment.

### AI Platform Functionality

**Figure 2.**
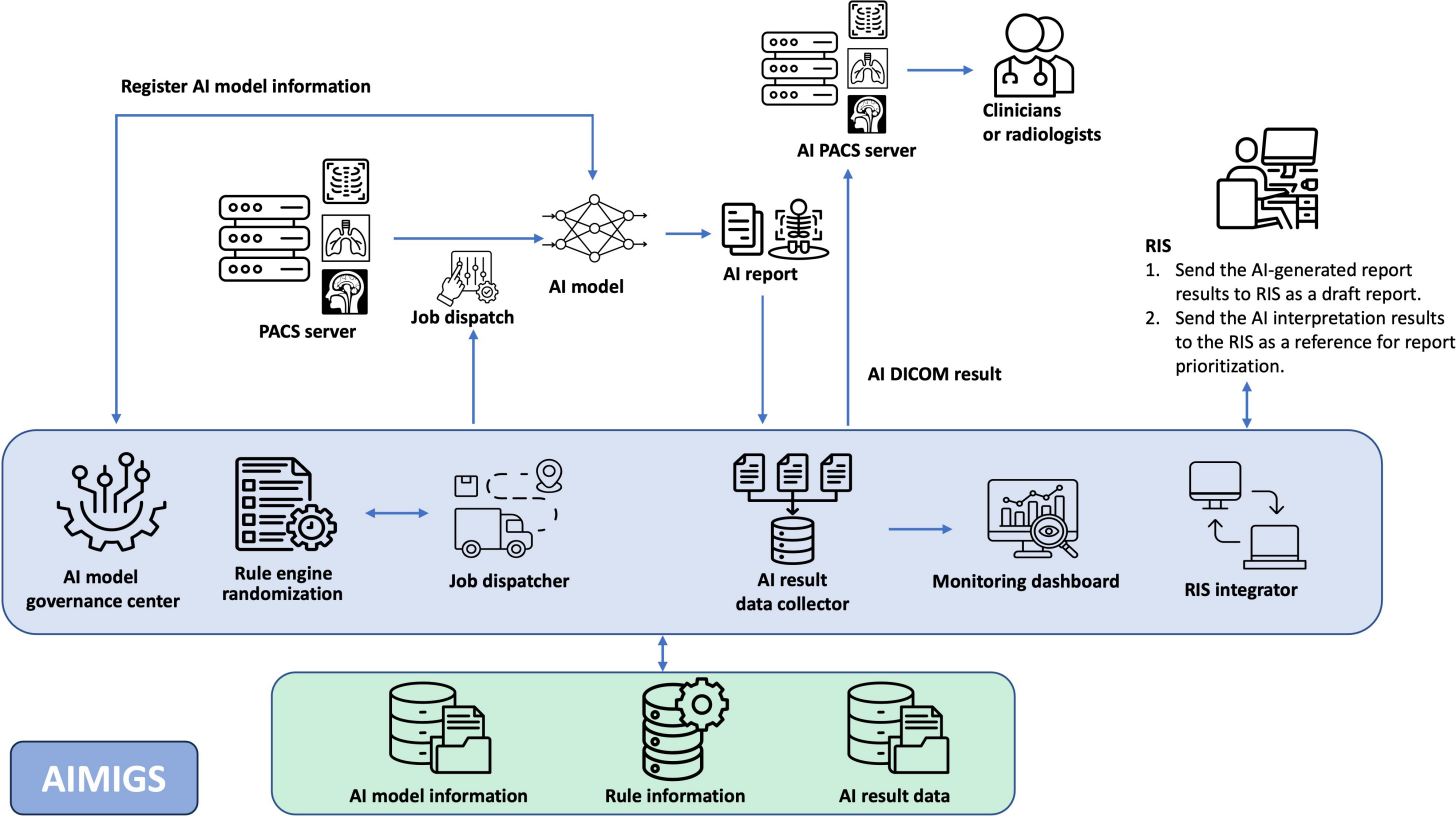
The randomization module within the AI Imaging Management, Integration, and Governance System (AIMIGS) rule engine. AI: artificial intelligence; PACS: Picture Archiving and Communication System; RIS: Report Information System.

**Figure 3.**
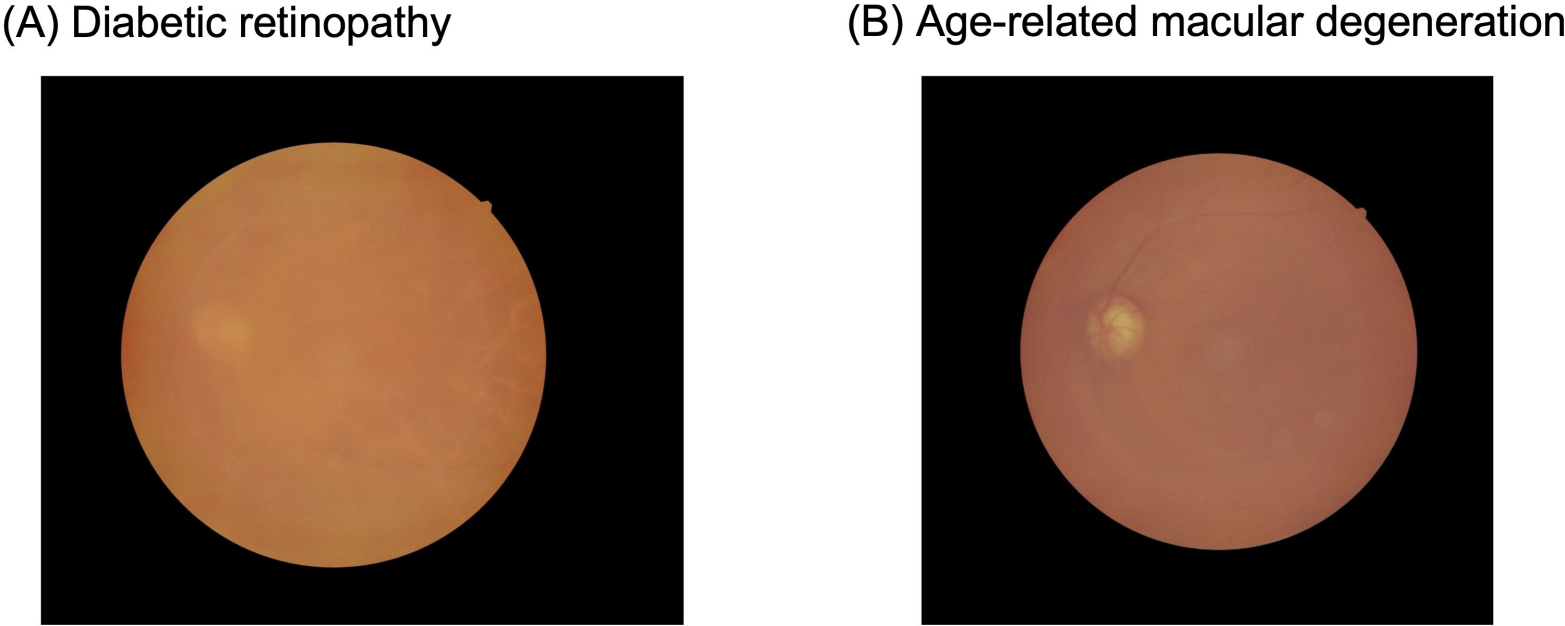
Artificial intelligence–assisted screening output indicating insufficient image quality. (A) Fundus image of diabetic retinopathy. (B) Fundus image of aAge-related Macular Degenemacular degenerraration.

AI-assisted retinal screening will be operationalized through the AI Imaging Management, Integration, and Governance System (AIMIGS), a centralized platform designed to govern, integrate, and deploy medical imaging–based AI models within routine clinical workflows (Figure 2). Within this trial, AIMIGS automatically routes eligible fundus photographs from the hospital Picture Archiving and Communication System (PACS) to the designated AI screening models (VeriSee DR and VeriSee AMD) according to predefined rules based on imaging modality, clinical order information, and participant allocation. AI-generated outputs, including image quality assessment, disease suspicion, and screening parameters, will be collected and returned to the AI PACS server and report information system, where they will be made available to treating physicians during clinical encounters. Clinicians are provided with AI-assisted outputs integrated into the clinical workflow. These include referral recommendations (positive or negative), disease severity grading for DR and AMD, image quality assessment, and lesion-level annotations where applicable. The AI results are presented through the electronic health record (EHR) system to support clinical decision-making. The AI system evaluates fundus images based on predefined technical criteria (eg, clarity, illumination, and visibility of key retinal structures), and images not meeting these criteria will be flagged as insufficient quality (Figure 3). Physicians may also independently identify images as inadequate for clinical interpretation. In such cases, repeat imaging will be performed. AIMIGS incorporates a rule engine module that enables automated and protocol-compliant random assignments.

When the input fundus image does not meet predefined quality criteria, the AI system classifies the image as inadequate for clinical interpretation and generates a notification indicating that the image quality is insufficient for analysis. In such cases, repeat image acquisition is required.

AIMIGS includes 3 primary storage components for managing AI model information, rule information, and AI result data, along with 6 functional modules: AI model governance, rule engine, job dispatcher, AI result data collector, monitoring dashboard, and Report Information System integrator. The randomization module is implemented as a core function within the rule engine. Following fundus photography acquisition, the original retinal images are transmitted to and stored in the PACS server. The job dispatcher then executes participant randomization according to the predefined rules in the rule engine and, based on the assigned study arm, determines whether DICOM images should be retrieved from the PACS server and forwarded to the VeriSee model for AI analysis or withheld from AI processing. AI-generated outputs are subsequently transmitted to the AI PACS server, and the AI-generated reports can be further integrated into the EHR as part of the clinical documentation.

In addition to supporting clinical trial randomization, AIMIGS serves as a comprehensive AI image management platform. Key modules include AI model governance for registration and lifecycle tracking; rule engine and task assignment for directing DICOM images to the appropriate AI models; results collection for storing images, reports, and diagnostic parameters; a monitoring dashboard to oversee performance and workflow; and an image-report integration module that facilitates report drafting, prioritization, and communication with the Radiology Information System or PACS.

### Randomization

Participants will be randomized at the individual level in a 1:1 ratio to either the AI-assisted screening group or the conventional physician-based screening group. Randomization is performed on a per-participant basis, and sample size estimation will be based on this process. DR and AMD are managed within a single unified cohort. DR and AMD can be diagnosed in the same patient, while DR and AMD are treated as separate disease-specific outcomes, and analyses will be conducted independently for each condition based on predefined eligibility criteria. Participants eligible for both conditions may contribute to both analyses.

In the conventional physician-based screening arm, fundus photographs will be interpreted by physicians without the use of any AI assistance. Physicians will review the images independently based on their clinical judgment. All physicians involved in image interpretation will receive standardized training in fundus photograph assessment prior to study initiation. In addition, a standardized reading guide will be provided to ensure consistency in image interpretation across the control group.

Randomization will be conducted using a computerized randomization module embedded within the rule engine of AIMIGS. After enrollment, each participant will be assigned a unique study ID, and allocation will be automatically generated by AIMIGS. Once the assignment is generated, the system automatically allocates each participant to the designated study arm and triggers the corresponding imaging procedures according to the predefined workflow.

### Intervention

The intervention consists of an AI-assisted retinal screening strategy using validated screening software (VeriSee DR and VeriSee AMD) for DR and AMD in routine outpatient care. The comparator consists of a conventional physician-based retinal screening strategy without AI assistance.

**Figure 4.**
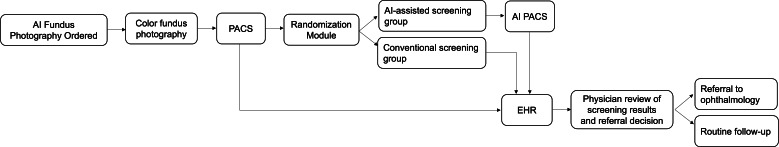
Workflow of artificial intelligence (AI)–assisted fundus screening integrated with Picture Archiving and Communication System (PACS) and electronic health record (EHR).

Participants assigned to the AI-assisted screening group will undergo color fundus photography as part of usual clinical care and will be screened using the AI-assisted approach. Treating physicians will be provided with AI-generated screening outputs, including image quality assessment, disease suspicion, and referral recommendations, in conjunction with fundus images during the clinical encounter. On the basis of this information, physicians will determine whether referral for confirmatory ophthalmologic evaluation is indicated. Participants assigned to the conventional screening group will undergo color fundus photography alone. Fundus images will be reviewed by treating physicians without AI assistance, and referral decisions will be made based solely on physicians’ independent clinical judgment (Figure 4).

The clinical workflow begins with the physician issuing an AI-assisted fundus photography order, followed by fundus photography acquisition and image upload to the PACS. The AI system then retrieves the images and triggers randomization via the AIMIGS system. In the experimental group, images undergo AI analysis, and the results are uploaded back to AI PACS. In the control arm, images are not analyzed by AI. Finally, physicians review the results in the EHR.

When a fundus image is considered insufficient for clinical interpretation, trained health care personnel will immediately reacquire the image on-site and reassess its quality. This process will not require any additional clinic visits or rescheduling. In both the AI-assisted and conventional screening arms, retinal imaging, image interpretation, and referral decisions are performed identically, ensuring that both groups follow the same care pathway at the same clinical time point. Both study arms follow an identical workflow. The only difference between groups is that the AI-assisted arm provides an additional AI-generated output to support physician decision-making, whereas the control arm relies solely on physician interpretation without AI assistance.

### Referral After a Positive Test

The examination will be repeated for images that are of insufficient quality for clinical interpretation. Across both groups, ophthalmology referral will be provided for participants with DR of stages 2 to 4 or AMD of stages 3 to 4, whereas participants not meeting these severity thresholds will be advised to undergo regular annual follow-up examinations. Confirmatory diagnosis of DR and AMD will follow the Early Treatment Diabetic Retinopathy Study [16], AREDS scales [23], and Beckman Initiative Classification [23] (Textbox 1). At 8 weeks after screening, referral completion and diagnostic outcomes will be assessed through review of electronic medical records, hospital visit records, and, when necessary, telephone follow-up.

Textbox 1. **Confirmatory diagnosis of diabetic retinopathy (DR) and age-related macular degeneration (AMD).**

Diabetic retinopathy

1. According to the Early Treatment Diabetic Retinopathy Study severity scale [24], primarily diagnosed through fundus photography and indirect ophthalmoscopy to identify typical features such as microaneurysms, retinal hemorrhages, hard exudates, and cotton-wool spots (soft exudates).
2. Fluorescein angiography is generally not used as a primary diagnostic tool; however, it may be used in the evaluation of macular ischemia, edema, or neovascularization to inform treatment strategies. It facilitates the verification of the location and extent of lesions.
3. The severity of DR is categorized into 5 stages: no diabetic retinopathy (no DR, stage 0), mild nonproliferative diabetic retinopathy (NPDR; stage 1), moderate NPDR (stage 2), severe NPDR (stage 3), and proliferative diabetic retinopathy (PDR; stage 4).

Age-related macular degeneration:

1. According to the Age-Related Eye Disease Study severity scale [25], fundus photography and indirect ophthalmoscopy are primarily used to identify the location, size, and number of drusen.
2. On the basis of the Beckman Initiative Classification [23], AMD is classified into 4 severity levels based on drusen size and pigmentary abnormalities: no AMD (stage 1), early AMD (stage 2), intermediate AMD (stage 3), and advanced AMD (stage 4).
3. When necessary, fluorescein angiography and optical coherence tomography are used to detect the choroidal neovascularization associated with AMD, the presence of macular edema, and retinal pigment epithelium abnormalities.

After completing the fundus photography, participants will be invited to complete a satisfaction survey regarding the screening method. Additionally, physicians participating in the study will be required to complete a satisfaction survey at the end of the trial, reflecting their experiences using the AI-assisted screening tool.

### Performance of AI-Assisted Ophthalmic Screening Tools

Both screening tools are based on the convolutional neural networks for image segmentation and classification of disease severities. Both tools received approval from the Taiwan Food and Drug Administration in 2020 (MOHW-MD-No.006966 and MOHW-MD-No.007652), with both classified as “Class II medical devices.” Once installed on a compatible computer, the software will automatically assess image quality and generate preliminary diagnostic outputs, which are immediately shared with attending physicians to support clinical decision-making. VeriSee DR uses deep learning algorithms to identify key DR features, including microaneurysms, hemorrhages, soft exudates, and hard exudates. The tool also offers referral recommendations, categorized as routine follow-up or ophthalmology referral.

**Figure 5.**
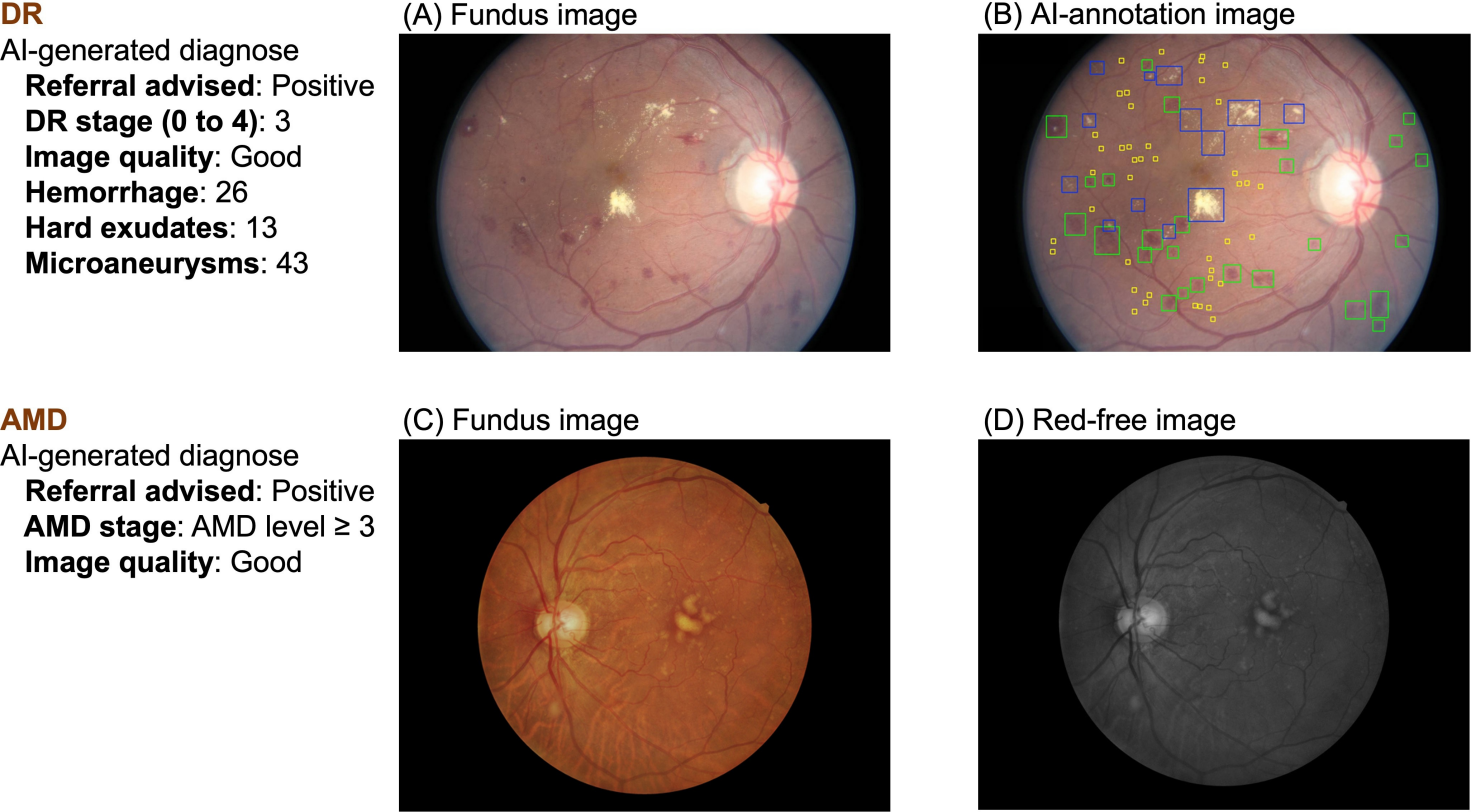
Demonstration of artificial intelligence (AI)–assisted screening for diabetic retinopathy (DR; A and B) and age-related macular degeneration (AMD; C and D).

The diagnostic accuracies of VeriSee DR and VeriSee AMD have been previously reported [13,26]. For DR, prior validation studies reported a sensitivity of 92.2% and a specificity of 89.5% [13]. For AMD, the results were 90% and 92.4%, respectively [26]. Figure 5 illustrates the AI-assisted screening interfaces for DR and AMD. For DR, the interface displays image quality assessment and AI-assessed severity staging with lesion-level annotations, whereas for AMD, the interface provides an AI-generated referral recommendation.

For DR screening, the AI system outputs the original color fundus image (panel A), a referral recommendation (positive or negative), DR severity grading (0: no DR; 1: mild nonproliferative diabetic retinopathy [NPDR]; 2: moderate NPDR; 3: severe NPDR; and 4: proliferative diabetic retinopathy [PDR]), image quality assessment (good or bad), and automated lesion annotation with lesion counts (panel B). As shown on the right, annotated lesions include hemorrhages (green), hard exudates (blue), and microaneurysms (yellow).

For AMD screening, the AI system provides the original color fundus image (panel C), a referral recommendation (positive or negative), AMD severity grading (AMD level <3 or AMD level ≥3), image quality assessment (good or bad), and corresponding red-free image (panel D) to enhance the contrast of specific retinal structures and lesions.

### Treatment

For DR, management depends on both DR severity and the presence of diabetic macular edema (DME). Mild to moderate NPDR without vision-threatening DME is typically monitored with periodic follow-up, usually every 6 to 12 months, depending on the risk. As the disease progresses to severe NPDR, closer monitoring is generally advised, and anti–vascular endothelial growth factor (anti-VEGF) therapy may be considered for selected patients. Once the disease progresses to PDR, prompt treatment is essential. While pan-retinal photocoagulation remains a standard treatment for PDR, intravitreal anti-VEGF injections have also become an alternative, offering visual benefits with less peripheral field loss. Anti-VEGF therapy is the first choice for center-involved DME with visual impairment, and DME can occur at any stage of DR. In PDR complicated by nonclearing vitreous hemorrhage and/or macula-threatening tractional retinal detachment, pars plana vitrectomy is often indicated. Managing AMD is similar to that of DR, with treatment strategies tailored to the disease stage. Early AMD focuses on modifying risk factors and regular monitoring. For intermediate AMD, antioxidant vitamins and minerals based on the AREDS2 formula, which replaces β-carotene with lutein and zeaxanthin, should be considered to lower the risk of progression [27]. In advanced AMD, especially the neovascular type, intravitreal anti-VEGF therapy is the standard treatment. These agents inhibit choroidal neovascularization, decrease exudation and retinal fluid, and often help stabilize or enhance visual acuity [28].

### Outcome Measures

All participants in both the intervention and control groups will undergo follow-up assessments and review of medical records to evaluate the outcomes of DR and AMD. The primary, secondary, and subsidiary outcomes are shown below.

#### Primary Outcome

The primary outcomes are the detection rate and PPV of DR and AMD screening.

Detection rate:


$$\frac{Number\;of\;people\;with\;the\;confirmatory\;diagnosis\;of\;AMD\;or\;DR}{Number\;of\;people\;who\;participate\;in\;screening}$$

PPV:


$$\frac{Number\;of\;cases\;the\;confirmatory\;diagnosis\;of\;AMD\;or\;DR}{Number\;of\;screened\;positive\;cases}$$

Detection rate is defined as the proportion of screened participants who receive a confirmatory diagnosis of DR or AMD among all participants who undergo screening. PPV is defined as the proportion of participants with a confirmatory diagnosis among those who test positive in the screening.

The intention-to-screen population will include all randomized participants, who will be analyzed according to their assigned group regardless of adherence to the screening or referral process. Missing outcome data, particularly those resulting from incomplete referral follow-up, will be addressed under the intention-to-screen principle and reflected in the detection rate. Specifically, participants who test positive but are not referred for confirmatory diagnosis will remain included in the denominator when calculating detection rates, although they will not contribute to the numerator. The per-protocol population will include participants who complete the screening and adhere to the predefined study procedures. Participants with incomplete referral follow-up who test positive but do not comply with referral for confirmatory diagnosis will be excluded from both the numerator and denominator when calculating the PPV.

#### Secondary Outcomes

The secondary outcome is the cost-effectiveness of AI-assisted screening compared with conventional screening, assessed using the incremental cost-effectiveness ratio, under the societal perspective. Cost-effectiveness analyses will incorporate both the direct and indirect medical costs related to screening, diagnostic evaluation, and treatment.

#### Exploratory Outcomes

Exploratory outcomes include (1) screening participation rate, assessed by the number of participants and nonparticipants; (2) referral completion rate, defined as the proportion of referred participants who completed ophthalmologic confirmatory evaluation within the prespecified 2-month follow-up period; (3) patient satisfaction, evaluated using a posttrial survey; (4) whether AI-assisted screening reduces clinical decision-making time and physician workload, evaluated using a posttrial survey; (5) time to diagnosis of DR or AMD; and (6) visual loss as the long-term outcome.

### Sample Size Estimation

The estimated prevalence of any DR (across stages 1-4) and AMD is 27% and 10%, respectively [29,30]. For this study, the sample size is calculated based on the assumption that 10% of patients with diabetes aged ≥20 years are diagnosed with clinically significant DR (stages 2 to 4) in the Department of Family Medicine and the Department of Geriatrics and Gerontology. Using AI-assisted diagnostic tools, the detection is assumed to increase to 15% [31].

In this real-world pragmatic setting, there was no prior evidence on the referral rates for eye disease screening in Taiwan. This study therefore assumed a referral rate of approximately 70% based on large-scale pragmatic screening trials of fecal occult blood testing and *Helicobacter pylori* stool antigen testing in Taiwan [20,21]. Accordingly, it is assumed that 70% of screen-positive individuals will be successfully referred to ophthalmologists for further examination and with a 2-tailed test, a type I error (α) of .05, and a type II error (β) of .10, the required sample size is 2612 participants (1306 per group). Approximately 870 participants are expected to be enrolled annually over an anticipated 3-year study period.

For patients aged ≥50 years in the Department of Family Medicine and the Department of Geriatrics and Gerontology in the NTUH, the prevalence of AMD is 5%, which is assumed to increase to 10% with the use of AI-assisted diagnostic tools. Assuming that 70% of the screening cases can be successfully referred to ophthalmologists and with a 2-tailed test, a type I error (α) of .05, and a type II error (β) of .10, the required sample size is 1650 participants (825 per group). Approximately 550 participants are expected to be enrolled per year.

### Data Security

After completing fundus photography, image files are automatically sent to the PACS for both the intervention and control groups or the AI PACS for only the intervention group. To protect patient privacy, secure the medical device and software, and maintain hospital information system stability, this study will apply to the Center of Intelligent Healthcare at NTUH for the “Trial Use of AI/Machine Learning Medical Device Software.” This step will verify that VeriSee DR and VeriSee AMD meet all relevant regulations, allowing their safe and effective use in clinical practice.

Study-related data will be managed using REDCap, a secure, web-based electronic data management system. REDCap will be used to record participant eligibility assessment, randomization assignments, physician interpretation results, and AI-generated screening outputs. Research data collected in REDCap will be stored separately from the hospital clinical information systems, with access restricted to authorized study personnel through role-based permissions. All data handling procedures will comply with institutional data protection policies and relevant data privacy regulations.

### Statistical Analysis

Baseline characteristics of the 2 study groups will be summarized as percentages for categorical variables and as means with SDs for continuous variables. Categorical variables will be compared using the chi-square test, and continuous variables will be compared using the 2-tailed *t* test. The primary analysis will follow the intention-to-screen principle, in which all randomized participants are analyzed according to their assigned study arm regardless of adherence to subsequent screening and management processes. Thus, participants will remain in the primary analysis regardless of whether AI interpretation is successfully completed, whether referral is achieved after a positive test result, or whether missing data occur within the screening workflow. These participants will still be included in the analysis of the primary outcome estimates, namely the detection rates. A per-protocol analysis will also be conducted, including only participants who adhered to the predefined study procedures, namely successful AI interpretation and, for those with positive test results, successful referral for further evaluation. Participants with any missing data during the process will be excluded from the per-protocol analysis. This population will be used to assess secondary outcome estimates, namely the PPVs. Between-group comparisons of primary and secondary outcomes will be performed using the 2 sample proportional tests.

Although the study adopted a multicenter pragmatic design, randomization was conducted at the individual level. The AI instruments are assumed to perform consistently across different settings. Therefore, the primary analyses of the primary outcomes will be conducted as crude analyses without adjustment for potential differences in baseline characteristics or workflow processes between the study groups. Nonetheless, hospital site effects, physician-level variation, image quality, referral completion, and missing confirmatory diagnoses may have been imbalanced between groups during study execution in this pragmatic setting and could potentially confound the primary analyses. Therefore, subsidiary analyses will be conducted at both the participant and physician levels using predefined subgroup analyses to examine the consistency of screening effects across primary outcomes. These subgroups will include study site, image quality (participants with and without poor-quality images for which AI could not generate a report despite repeat testing), referral timeliness (with delayed referral defined as no referral within 2 mo), physician specialty (including family medicine and geriatric or gerontology care physicians), clinical setting (across the 4 participating hospitals), and participant-level characteristics (including age, sex, and comorbidities). These subsidiary analyses are exploratory in nature, and results will be presented descriptively without adjustment for multiple comparisons.

In addition to the originally planned 2-sample proportion comparisons in the randomized trial and subsidiary stratified analyses, post hoc multivariable regression models will be performed to account for potential clustering and confounding factors and to support the findings from the primary analyses. Site-level variability and physician-level effects will be addressed using mixed-effects models, with study site and/or physician included as random effects where appropriate. Adjustment for relevant covariates, including baseline characteristics of patients, image quality, referral completion status, and missing confirmatory diagnoses, will also be performed. Effect estimates will be reported as adjusted odds ratios with corresponding 95% CIs.

In addition, regarding the incomplete referral follow-up and missing confirmatory diagnoses in the pragmatic setting, the number and proportion of participants with missing outcome data will be summarized by study group, together with the reasons for missingness whenever available. The primary detection rate analysis will follow the intention-to-screen principle, in which screen-positive participants with missing confirmatory diagnosis will remain in the denominator but will not contribute to the numerator. For PPV analyses, participants with missing confirmatory diagnosis will be excluded from both the numerator and denominator. Post hoc mixed-effects regression analyses will be performed for these 2 outcomes accounting for other covariates as mentioned earlier.

A 2-sided *P* value of <.05 will be considered statistically significant. All statistical analyses will be conducted using the SAS software (version 9.4; SAS Institute).

### Cost-Effectiveness Analysis

**Figure 6.**
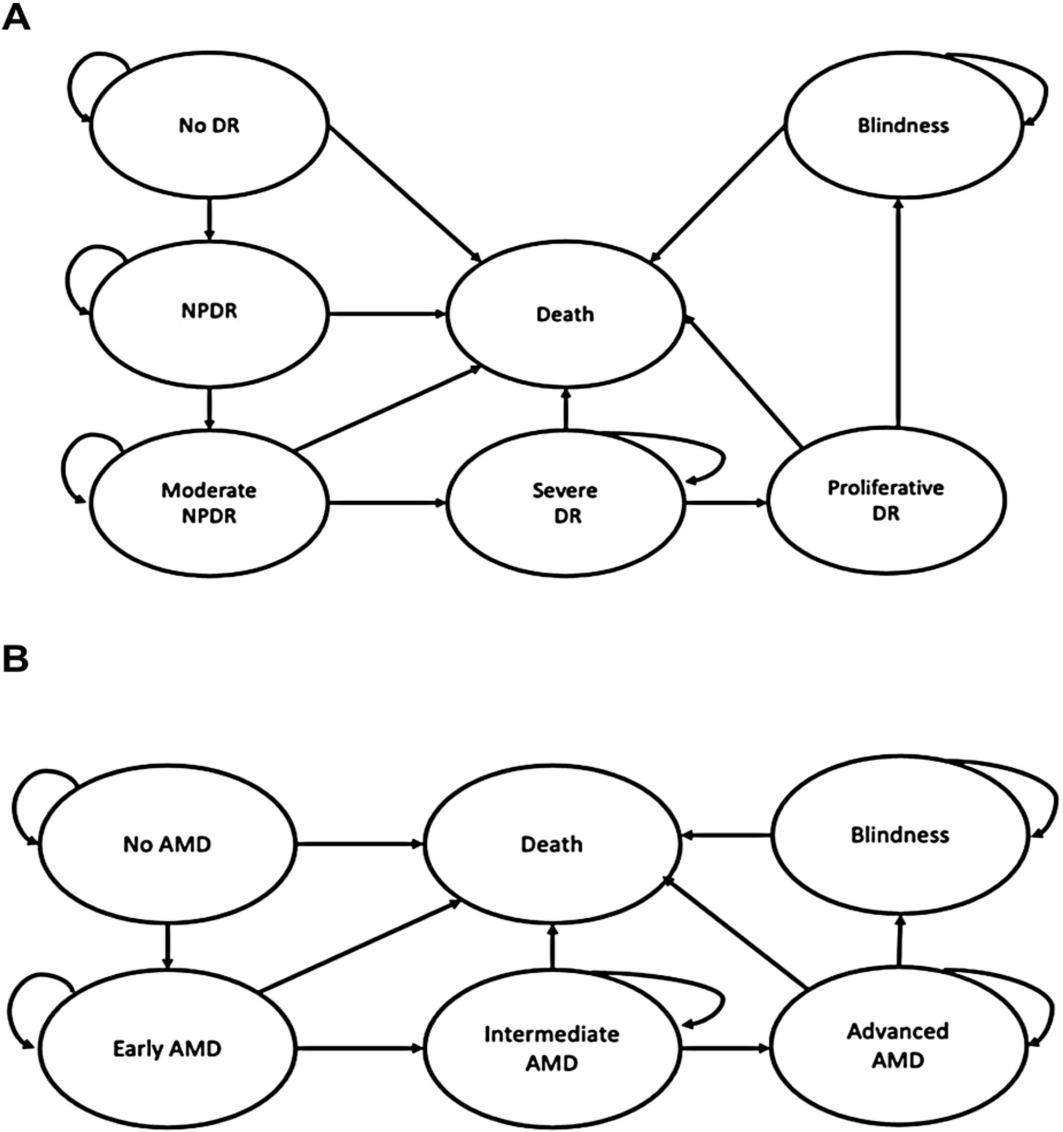
Markov model structure. (A) The diabetic retinopathy (DR) Markov model consists of 7 mutually exclusive health states: no DR, mild nonproliferative diabetic retinopathy (NPDR), moderate NPDR, severe NPDR, proliferative DR, blindness, and death. Disease progression is assumed to be irreversible, with transitions allowed only from less severe to more severe states or remaining in the same nonabsorbing state (self-loops). Death represents all-cause mortality. (B) The age-related macular degeneration (AMD) Markov model includes 6 mutually exclusive health states: no AMD, early AMD, intermediate AMD, advanced AMD, blindness, and death. Transitions are assumed to be unidirectional, allowing progression to more advanced disease stages or remaining in the current state. Death represents all-cause mortality.

**Figure 7.**
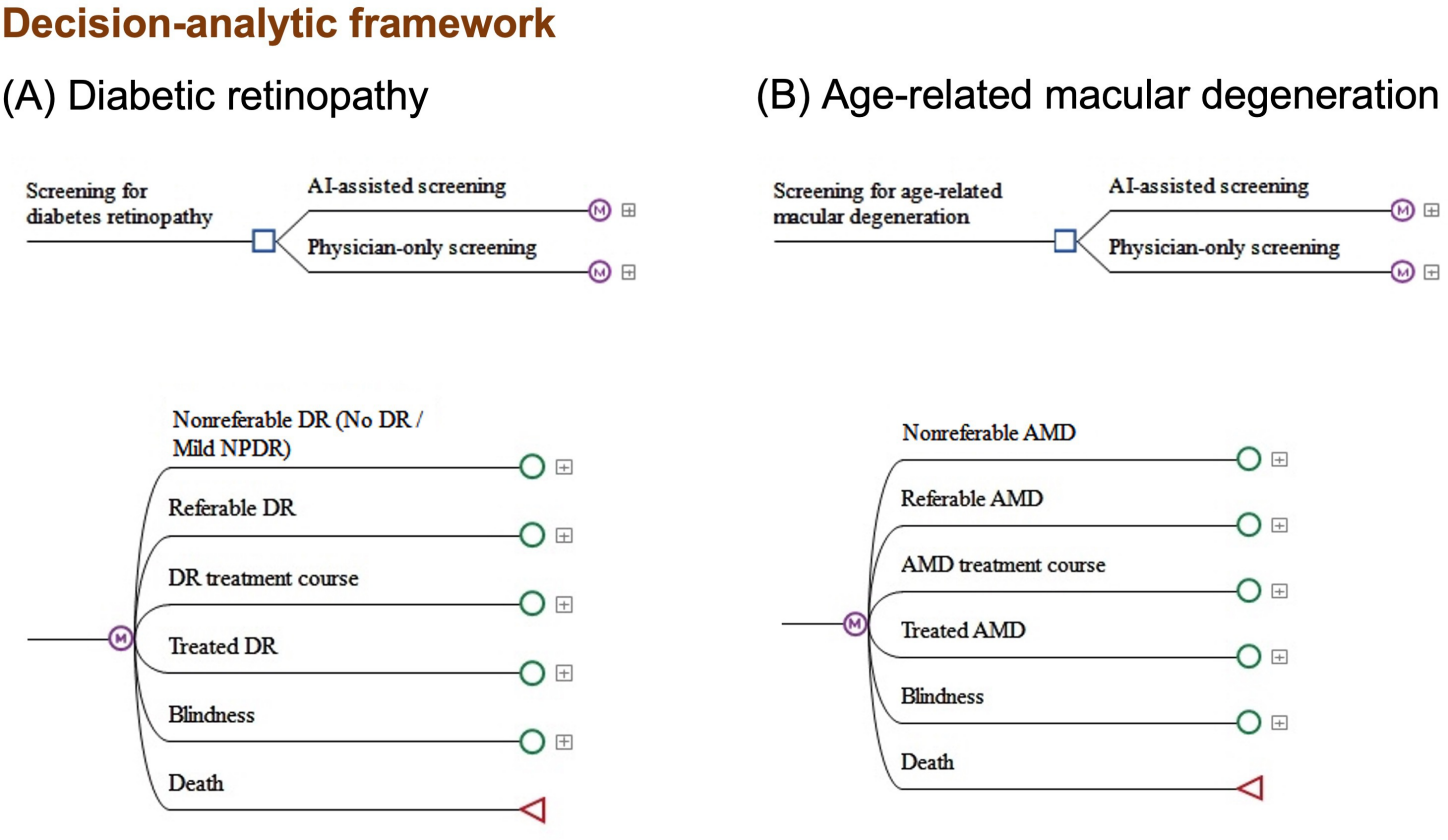
Decision-analytic framework for (A) diabetic retinopathy (DR) and (B) age-related macular degeneration (AMD) illustrating the decision tree structure for screening outcomes followed by downstream disease progression pathways under artificial intelligence (AI)–assisted and conventional screening strategies. NPDR: nonproliferative diabetic retinopathy.

As long-term outcomes, such as blindness, require extended follow-up, their assessment is impractical, given the rapid evolution of AI-assisted diagnostic software and the frequent release of updated versions throughout the product life cycle. Therefore, a model-based cost-effectiveness analysis will be conducted to compare the AI-assisted screening strategy with conventional physician-based screening for DR and AMD. Clinical effectiveness inputs, including screening detection rates and referral outcomes, will be derived from the randomized trial. These natural course models are illustrated in Figure 6A (DR) and Figure 6B (AMD). The decision analysis will include two arms following the decision node: (1) usual care and (2) the AI-assisted model. A decision-analytic framework incorporating both a decision tree and a Markov model will be developed to simulate disease progression, downstream clinical management, and long-term health outcomes following screening (Figure 7). Health states will represent different stages of disease progression for DR and AMD, as well as blindness and death; therefore, extending the analysis beyond the eye disease screening process primarily evaluated in this trial.

At the screening stage, individuals will be classified as true positives, false positives, true negatives, or false negatives based on the diagnostic performance of each strategy. Patients identified as positive will be referred for confirmatory diagnosis and subsequent treatment, whereas those with negative results will not undergo further evaluation at that time. Individuals with false-negative results are assumed to experience delayed diagnosis and will follow the natural history of disease progression until clinical detection. Transition into the Markov model is determined by the initial screening outcomes, and health outcomes and costs associated with disease progression, vision impairment, and treatment will be accumulated over the analytic time horizon.

For DR, disease progression will be modeled using a Markov framework based on established natural history models [32-34]. Health states will include no DR, nonproliferative DR (mild, moderate, and severe), proliferative DR, blindness, and death. For AMD, health states will include no AMD; early, intermediate, and advanced AMD; blindness; and death. In both models, health states are mutually exclusive and collectively exhaustive. Disease progression is assumed to be irreversible, allowing transitions only from less severe to more advanced disease stages or remaining in the same nonabsorbing state (self-loops).

Patients with false-negative results (ie, individuals with DR or AMD not detected through screening) are modeled as following the natural history of disease progression until clinical detection, reflecting the impact of delayed diagnosis on both health outcomes and associated medical costs for treatment.

Cost estimation will be conducted from both the societal perspective and the National Health Insurance (NHI) payer perspective, a single-payer system. Costs considered will include direct medical costs (screening, follow-up visits, and treatments), indirect medical costs (information technology maintenance and health care workforce resources), and indirect costs (transportation and productivity loss due to visual impairment). The model will include screening costs, follow-up diagnostic costs, and direct medical costs associated with the diagnosis and treatment of DR and AMD. From the societal perspective, indirect costs, including transportation and productivity loss due to visual impairment, will also be incorporated.

All costs will be estimated in New Taiwan Dollars and, if applicable, converted to US dollars based on the average exchange rate for the study year of 2026. Cost parameters will be primarily derived from the Taiwan NHI reimbursement schedule [35], supplemented by institutional data. All these data will be obtained from the NTUH-integrated Medical Data Center [36].

As AI-assisted screening has not yet been reimbursed under the Taiwan NHI, the cost of AI-assisted screening will be estimated based on assumptions and expert consultation. Screening and follow-up diagnostic costs will be based on standard clinical practice and reimbursement policies under the NHI system. Treatment costs for DR and AMD will be estimated using a combination of NHI fee schedules and published sources. Where necessary, assumptions will be informed by expert opinion. After screening, individuals with true-positive results will incur costs related to screening, confirmatory diagnostic evaluation, and subsequent treatment. Those with false-positive results will incur screening and unnecessary referral costs, including additional ophthalmology visits and diagnostic procedures. True-negative cases will be assumed to incur screening costs only, with no further downstream costs. For individuals with false-negative results, costs will include initial screening costs as well as additional downstream costs associated with delayed diagnosis, disease progression, and subsequent treatment at more advanced stages. These cases may also incur higher long-term costs related to vision impairment and productivity loss. Costs and quality-adjusted life years (QALYs) will be discounted at 3%.

Quality-adjusted life years will be used to quantify health benefits and evaluate cost-effectiveness. QALYs integrate survival and health-related quality of life by weighting life years with health state utility values. In this study, QALYs will be calculated by multiplying the expected survival years by corresponding utility weights for each health state, with values ranging from 0 (blindness) to 1 (perfect health); negative values may be applied to health states perceived as worse than blindness.


QALY=years of life×health state weight

The primary outcome of the cost-effectiveness analysis is the incremental cost-effectiveness ratio, defined as the difference in costs divided by the difference in effectiveness between the 2 screening strategies. Given the limited duration of trial follow-up, a model-based economic evaluation using a Markov model will be conducted to project long-term costs and outcomes following screening. Detection rate will serve as the primary effectiveness measure.


$$ICER=\frac{\Delta cost}{\Delta effectiveness}=\frac{cost_{new}-cost_{old}}{effectiveness_{new}-effectiveness_{old}}=\frac{Incremental\;Cost\;}{\;incremental\;effectiveness\;}\;\left(common:cost\;per\;QALY\right)$$

To assess parameter uncertainty, one-way sensitivity analyses and probabilistic sensitivity analyses will be performed. Results will be summarized using cost-effectiveness planes and cost-effectiveness acceptability curves. The analyses will be performed using TreeAge Pro 2024 (TreeAge Software, Inc).

### Ethical Considerations

The study was approved by the Research Ethics Committee of NTUH (reference 202505015DINE, version 5.0; approved on October 28, 2025), Fu Jen Catholic University Hospital (FJUH114483), and Min-Sheng General Hospital (CIRB2025003). Investigators retained full control of the protocol, database, analysis, and publication. The software company (Acer) will not be able to access identifiable or raw study data. The company was not involved in the design of the study protocol or the decision to submit this manuscript for publication. The company will not have access to the study data during study execution and will not be involved in participant recruitment, data collection, data management, statistical analysis, interpretation of the results, or further manuscript preparation.

### Quality, Safety, and Monitoring

The AI-assisted tools perform no invasive interventions on participants, and the analysis is conducted solely based on data collected during the study. The output will not be used for diagnosis and is considered a nonsignificant risk AI-based or machine learning–based software as a medical device. Furthermore, this study is noninvasive and does not involve the use of mydriatic agents for fundus photography, thus minimizing any potential iatrogenic harm to participants. Given the low-risk and noninterventional nature of the study, an independent Data Monitoring Committee will not be established. Instead, study oversight and safety monitoring will be conducted by the principal investigators and the study steering team, who will periodically review study conduct, data integrity, and any unexpected safety-related issues on a quarterly basis throughout the trial.

## Results

The VeriSee AI-assisted screening tools aid physicians in the real-time interpretation of retinal images, thereby decreasing their workload across hospital departments and reducing the duration required for patients to receive their results. This method enhances the practicality and acceptance of ophthalmic screening within routine clinical environments. Data collection commenced in October 2025 and is currently ongoing. It is anticipated that AI-assisted screening may improve detection rates and PPV for DR and AMD by facilitating earlier identification of clinically relevant abnormalities and more appropriate referral decisions, particularly among individuals with diabetes and older adults. Earlier detection and referral may enable timely intervention, which is a key factor in preventing vision impairment. From a health system perspective, improved screening efficiency and more targeted referrals may contribute to favorable cost-effectiveness by optimizing resource use and reducing unnecessary ophthalmology visits. The findings of this study may provide robust evidence to inform future evaluations of AI-assisted retinal screening within the context of NHI coverage, including considerations of clinical effectiveness, feasibility, and economic value.

## Discussion

### Principal Results

This study protocol describes an AI clinical trial that evaluates the efficacy of 2 AI-assisted screening tools in enhancing the detection and referral processes for DR and AMD within clinical practice. Using an RCT design, participants will be assigned to 2 groups. The intervention group will incorporate AI-based image analysis into routine medical procedures, providing preliminary screening outputs to assist physicians in clinical decision-making, with feedback provided to participants in a timely manner. Conversely, the control group will use conventional eye disease screening procedures, with results reviewed and communicated during follow-up appointments. Through direct comparison of AI-assisted and conventional screening pathways, this study will assess whether the integration of AI-based tools is associated with differences in referral patterns, screening efficiency, and timeliness of clinical decision-making in routine care settings. In particular, the trial will examine the potential of AI-assisted screening to support more appropriate ophthalmology referrals and reduce delays within existing clinical workflows. The long-term outcomes will be mainly simulated using the decision tree analytic model. In addition, patient- and physician-reported satisfaction data will be collected to evaluate the feasibility, acceptability, and perceived usefulness of AI-assisted screening in real-world clinical settings. Together, these findings are expected to provide empirical evidence on the role of AI-assisted retinal screening in supporting more efficient, patient-centered, and digitally enabled ophthalmic care.

### Limitations

Although this study is designed as a low-risk pragmatic clinical trial, several limitations should be acknowledged. First, while detection rate and PPV provide meaningful indicators of real-world screening effectiveness and downstream care engagement, they do not capture all dimensions of screening accuracy or long-term patient benefit. Important outcomes such as false-negative rates, disease progression, visual outcomes, and treatment-related outcomes are not designated as central end points in this protocol. These outcomes represent important areas for future investigation beyond the scope of the current pragmatic trial. Nonetheless, these will be estimated using the simulation models. Second, follow-up data may be incomplete for participants who seek subsequent ophthalmologic care outside the NTUH health care system, Fu Jen Catholic University Hospital, and Min-Sheng General Hospital. In such cases, confirmatory diagnostic results and downstream clinical outcomes may not be fully captured, potentially limiting the completeness of outcome assessment. This issue will be evaluated in the subgroup analyses. Third, the AI-assisted screening tools evaluated in this study are designed to detect specific retinal conditions only, including the DR and AMD. Other ocular diseases or retinal pathologies are not within the diagnostic scope of these tools and therefore may not be identified through the AI screening process while they may be identified by physicians. These will be evaluated in the post hoc analyses. Fourth, suboptimal image quality and inherent limitations in AI-based interpretation may result in false-positive or false-negative screening results, which could lead to unnecessary referrals or missed cases. To reduce this risk, image quality will be monitored at the time of acquisition, with repeat imaging performed when necessary, and cases with uncertain findings will still be referred for ophthalmologic evaluation. The impact will be evaluated in the subgroup or post hoc analyses. Fifth, real-world health care system constraints may influence the effectiveness of the screening-to-referral pathway. Limited ophthalmology workforce capacity and outpatient clinic availability may affect referral completion and time to confirmatory diagnosis, particularly in high-volume clinical settings. These constraints reflect routine clinical practice and may impact the generalizability of study findings to health care systems with different resource availability. Finally, it is important to emphasize that the AI-assisted screening tools are intended to support, rather than replace, clinical judgment. Treating physicians and ophthalmologists retain full responsibility for diagnostic confirmation and treatment decisions. Medical personnel involved in the study will receive training on appropriate interpretation and use of AI-assisted outputs to ensure safe and effective integration into clinical care.

### Conclusions

This study aims to evaluate the clinical effectiveness and feasibility of incorporating AI-assisted retinal screening tools into routine practice. By comparing AI-assisted and traditional screening methods, the trial will generate evidence on whether these tools improve early detection, enhance referral efficiency, and increase patient and physician satisfaction. The results are expected to guide the broader adoption of AI technologies in ophthalmic care, leading to more accessible, timely, and precision-oriented eye health services.

## Supplementary material

10.2196/91699 Checklist 1 SPIRIT 2025 editable checklist.

## Abbreviations

AI: artificial intelligence
AIMIGS: AI Imaging Management, Integration, and Governance System
AMD: age-related macular degeneration
anti-VEGF: anti–vascular endothelial growth factor
DME: diabetic macular edema
DR: diabetic retinopathy
EHR: electronic health record
NHI: National Health Insurance
NPDR: nonproliferative diabetic retinopathy
NTUH: National Taiwan University Hospital
PACS: Picture Archiving and Communication System
PDR: proliferative diabetic retinopathy
PPV: positive predictive value
QALY: quality-adjusted life years
RCT: randomized controlled trial
SPIRIT: Standard Protocol Items: Recommendations for Interventional Trials
